# Type H vessels: functions in bone development and diseases

**DOI:** 10.3389/fcell.2023.1236545

**Published:** 2023-11-16

**Authors:** Xiaonan Liu, Peilin Zhang, Yuan Gu, Qiaoyue Guo, Yonggan Liu

**Affiliations:** ^1^ Department of Orthopedics, Shanghai Sixth People’s Hospital Affiliated to Shanghai Jiao Tong University School of Medicine, Shanghai, China; ^2^ Department of Colorectal and Anal Surgery, Zhongshan City People’s Hospital, Zhongshan, Guangdong, China; ^3^ Endocrinology Research Center, Department of Endocrinology, Xiangya Hospital of Central South University, Changsha, Hunan, China

**Keywords:** type H blood vessel, bone development, bone formation, osteogenesis, bone disease, osteoblast, VEGF, PDGF-BB

## Abstract

Type H vessels are specialized blood vessels found in the bone marrow that are closely associated with osteogenic activity. They are characterized by high expression of endomucin and CD31. Type H vessels form in the cancellous bone area during long bone development to provide adequate nutritional support for cells near the growth plate. They also influence the proliferation and differentiation of osteoprogenitors and osteoclasts in a paracrine manner, thereby creating a suitable microenvironment to facilitate new bone formation. Because of the close relationship between type H vessels and osteogenic activity, it has been found that type H vessels play a role in the physiological and pathological processes of bone diseases such as fracture healing, osteoporosis, osteoarthritis, osteonecrosis, and tumor bone metastasis. Moreover, experimental treatments targeting type H vessels can improve the outcomes of these diseases. Here, we reviewed the molecular mechanisms related to type H vessels and their associated osteogenic activities, which are helpful in further understanding the role of type H vessels in bone metabolism and will provide a theoretical basis and ideas for comprehending bone diseases from the vascular perspective.

## Introduction

The skeletal system supports the structure of the body and contributes to motor function. After the basic structure of the bone is formed, the skeletal system will succeed with the osteoclast–osteoblast bone remodeling process to adapt to the changing external mechanical environment, so the osteogenic ability is very important to maintain the function of the skeletal system (Langdahl et al., 2016). Studies have shown that neovascularization is closely related to osteogenic activity: neovascularization not only directly provides oxygen, nutrients, and growth factors for osteogenesis-active areas but also shapes the microenvironment suitable for the survival of mesenchymal stem cells and pre-osteoblasts through the secretion of angiocrine signals, thereby promoting osteogenic activity (Sivaraj and Adams, 2016). So far, three types of capillaries have been found to exist within bone tissue: types H, L, and E. Each type has different molecular markers, morphologies, locations, and unique functions (Kusumbe et al., 2014; Langen et al., 2017). Recently, by light-sheet confocal microscopy, a new kind of endothelial cell, namely, the lymphatic endothelium (LEC), was found to exist within the bone marrow (Biswas et al., 2023). It was found that bone marrow LEC was regulated by IL-6 through VEGF-C/VEGFR-3 signaling and genotoxic stress (Biswas et al., 2023). These results demonstrated the highly diversified nature of bone marrow transport networks.

Type H blood vessels, a vascular subtype discovered in recent years, have been found to be closely associated with osteogenic activity. The H (high) blood vessels are distinguished by their elevated expression of endomucin (EMCN) and platelet endothelial cell adhesion molecule-1 (PECAM-1/CD31) (Emcn^High^ CD31^High^). In morphology, type H vessels develop as straight columns connected by vascular rings or arches. Studies have indicated that type H vessels are encompassed by a substantial quantity of pre-osteoblasts that express osteogenesis-specific transcription factors, namely, osterix and Runx2 (Kusumbe et al., 2014). There exists a reciprocal regulatory relationship between type H vessels and diverse cell populations within the bone marrow. Bone has the ability to modulate the angiogenesis process through the secretion of various factors such as vascular endothelial growth factor (VEGF), platelet-derived growth factor type BB (PDGF-BB), and slit guidance ligand 3 (SLIT-3). Vascular cells can influence the function of osteoblasts or osteoclasts by releasing factors like VEGF and Noggin (Wan et al., 2010). The aforementioned spatial co-localization and mutual regulation of neovascularization and osteogenesis are referred to as the coupling of angiogenesis and osteogenesis activities (Lafage-Proust et al., 2010). Numerous reports have shown that type H vessels have a significant impact on the processes of fracture healing, osteoporosis, osteoarthritis, osteonecrosis, tumor bone metastasis, and other bone-related disorders (Wan et al., 2010).

This article primarily focuses on the recent advancements in the study of type H vessels, specifically addressing their formation and regulatory mechanisms, as well as their significance in bone development and bone-related disorders.

### The role of type H vessels in bone formation and bone remodeling

The formation and maintenance of long bones include two distinct processes, namely, bone modeling and bone remodeling. The former term primarily pertains to the osteogenic activities involved in the transformation from a boneless state to the formation of the bone during skeletal development. On the other hand, the latter term refers to the localized process of bone reconstruction that occurs in response to external mechanical or metabolic factors after bone formation, commonly known as “remodeling” (Langdahl et al., 2016).

Studies have shown that neovascularization acts in both bone modeling and bone remodeling processes (Percival and Richtsmeier, 2013). During the process of bone modeling, the hypertrophic chondrocytes located beneath the growth plate secrete VEGF, which serves to attract vascular invasion toward the growth plate and leads to the recruitment and differentiation of osteoblasts. This process subsequently results in the mineralization of the area and the elongation of long bones (Maes et al., 2002). Kusumbe et al. found that these blood vessels were characterized by high expression of Emcn and CD31, hence were named type H (high) blood vessels (Kusumbe et al., 2014). In bone development, type H vessels proliferate actively and form abundantly within the cancellous bone region, periosteum, and endosteum of the long bone (Wan et al., 2010). In the cancellous bone region of long bones, interconnected vessel columns of type H vessels have been observed in adolescent mice. Oxygenated arterial blood is conveyed through arteries and enters type H vessels, which then traverse the blood sinuses in the diaphysis and finally drain into the venous system (Sivaraj and Adams, 2016). The observation of a substantial population of pre-osteoblasts expressing osterix and Runx2 in close proximity to type H vessels indicates a potential link between the development of these vessels and osteoblast function (Langen et al., 2017). In addition to osteoblasts, Romeo et al. recently discovered that a new specialized vascular-associated osteoclast (VAO) can promote the release of metalloproteinase-9 (MMP-9) from type H vessels (Romeo et al., 2019). This release of MMP-9 results in the degradation of cartilage tissue, consequently creating an environment that promotes the invasion of type H vessels in growth plates (Romeo et al., 2019). The findings indicate a significant correlation between the activity of osteoblasts and osteoclasts, and the presence of type H vessels. During the process of bone remodeling, Xie et al. unveiled that pre-osteoclasts possess the capability to induce the development of type H vessels in the active region of osteoclasts by means of secreting PDGF-BB (Xie et al., 2014). The perivascular cells of type H vessels also express mesenchymal stem cell markers such as PDGFRβ, nestin, and NG2. When bone is injured, these cells can transform into pre-osteoblasts through the activated Wnt pathway and promote bone repair (Chen et al., 2020; Matsushita et al., 2020).

### The regulation of type H blood vessels

**FIGURE 1:**
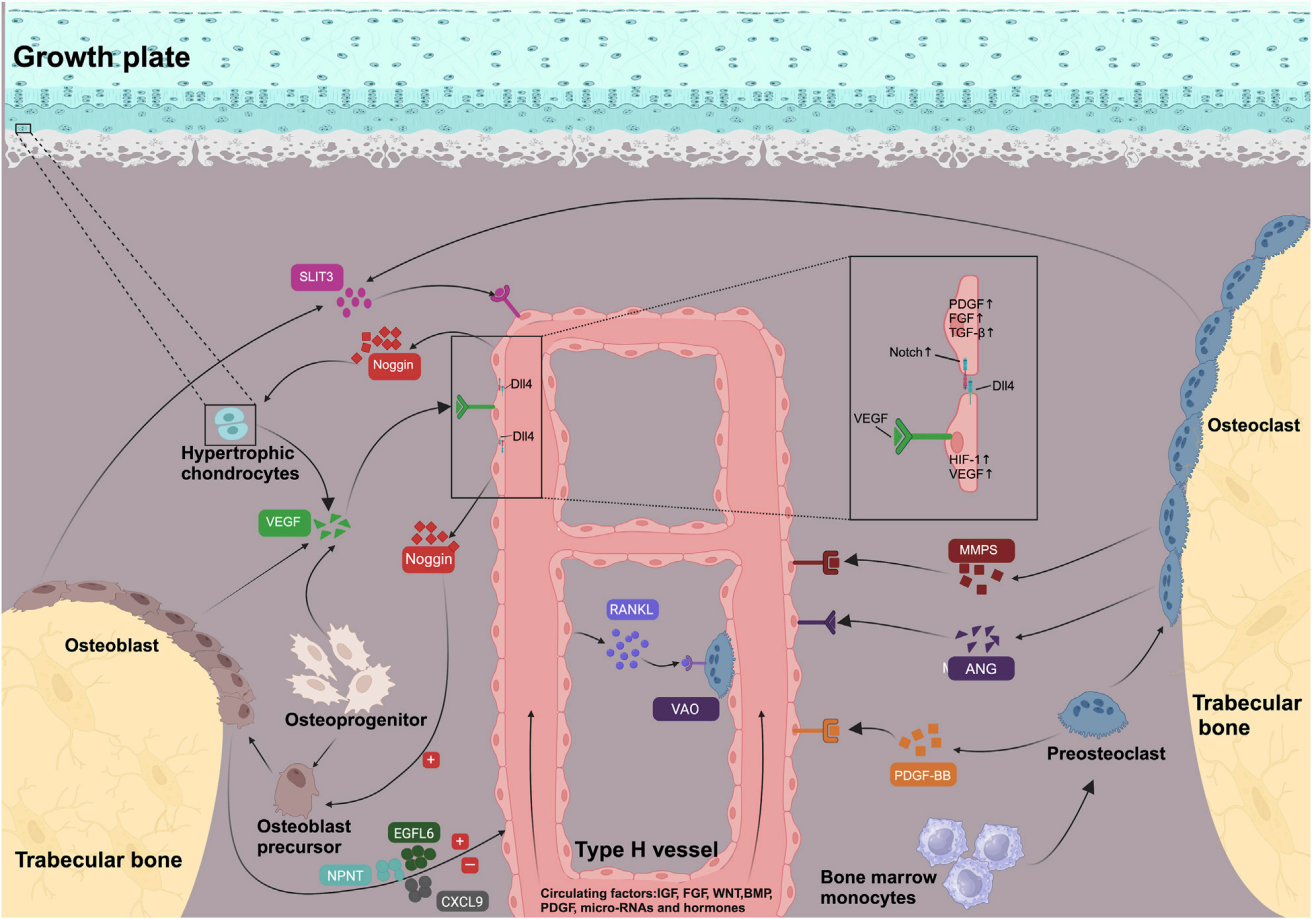
Signaling pathways and molecules involved in the development and maintenance of type H vessels and coupled osteogenesis. VEGF, PDGF-BB, SLIT-3, MMPs, and ANG secreted by bone cells in metaphysis support type H vessel formation. Osteoblasts secrete epidermal growth factor-like protein 6 (EGFL6) and nephronectin (NPNT) and chemokine (C-X-C motif) ligand 9 (CXCL9) to regulate angiogenesis. Regulated by HIF-1α under hypoxic conditions, VEGF activates the Notch pathway of the tip cells by increasing the Notch ligand DLL4, producing stalk cells and completing the process of angiogenesis. Type H vessels were surrounded by osteoprogenitors and promote matrix mineralization. Endothelial Notch/Dll4 signaling upregulated secretion of Noggin, which supports osteoblast differentiation and cartilage maturation. Meanwhile, type H vessels express RANKL and induce differentiation of VAO, which facilitates growth plate resorption and bone formation. Type H vessels secrete PDGF, FGF, and TGFβ to regulate adjacent bone cells or endothelial proliferation. Type H vessels were also regulated by systematic signaling including IGF, FGF, WNT, BMP, PDGF, micro-RNAs, and hormones (e.g., estrogen).

The link between type H blood vessels and osteogenic activity is based on the communication between vascular endothelial cells and osteoblasts or osteoclasts (Figure 1).

#### HIF-1α

The hypoxia-induced factor (HIF) is a crucial transcription factor involved in cellular oxygen sensing (Riddle et al., 2009). HIF is composed of a β-subunit and one of three α-subunits (HIF-1α, HIF-2α, and HIF-3α) (Peng et al., 2020). Under hypoxic tissue conditions, HIF-1α, expressed by osteoblasts and vascular endothelial cells, upregulates the expression of VEGF, thereby facilitating and promoting the formation of tissue neovascularization (Wan et al., 2010). Kusumbe et al. discovered that the levels of HIF-1α were increased in type H vessels within the bone marrow of adolescent mice. As mice aged, the levels of HIF-1α gradually decreased, which was accompanied by a reduction in type H vessels and a loss of bone mass. Activation of HIF-1α in vascular endothelial cells has been shown to enhance the proliferation of type H vessels in the bone marrow and increase the bone density (Kusumbe et al., 2014). The specific knockout of HIF-1α in vascular endothelial cells resulted in a decreased number of pre-osteoblasts and led to osteoporosis (Wan et al., 2010). Additionally, the HIF-1α pathway plays a regulatory role in osteoblast activity. Wan et al. conducted a study which demonstrated that the overexpression of HIF-1α in osteoblasts has the potential to enhance both angiogenesis and osteogenesis (Wan et al., 2010). Deferoxamine mesylate is an iron chelator that increases the activity and stability of intracellular HIF-1α. Researchers revealed that the use of deferoxamine mesylate can increase the number of osteoblasts by upregulating the number of type H vessels, thereby promoting osteogenesis (Jones and Harris, 2006). It is important to note, however, that this particular effect of HIF-1α on osteogenesis may be restricted to young mice. In adult mice, the deletion of HIF-1α in osteoblasts or osteocytes has been shown to enhance osteoblast activity and promote an increase in bone mass in response to mechanical loading (Riddle et al., 2011).

#### VEGF

The VEGF family in mammals consists of five protein subtypes (VEGFA, B, C, D, and PlGF (placental growth factor)), three receptors (VEGFR1, R2, and R3), and two co-receptors (neuropilin-1 and -2) (Grunewald et al., 2010; Koch, 2012). Among these subtypes, VEGFA is considered the most potent angiogenic growth factor (Helmrich et al., 2013). It primarily binds to the VEGFR2 receptor, thereby promoting the proliferation, migration, maturation, and survival of vascular endothelial cells. VEGF in the bone marrow originates from various sources. In the long bone, chondrocytes, osteoblasts, osteoclasts, immune cells, and perivascular cells were all proven to secrete VEGFA (Hu and Olsen, 2017). Gerber et al. demonstrated that hypertrophic chondrocytes play a crucial role in the process of endochondral ossification by promoting the infiltration of blood vessels into the cartilage region through the secretion of VEGF, thereby facilitating osteogenesis (Gerber et al., 1999). Maes et al. discovered that the deficiency subtypes of VEGF, namely, VEGF188 and VEGF164, resulted in decreased vascular invasion into cartilage and a reduction in bone marrow blood vessels. This ultimately led to limb shortening and osteoporosis (Maes et al., 2002). The knockout of the VEGFA or VEGFR gene in pre-osteoblasts resulted in osteogenic disorders and decreased osteogenic differentiation, suggesting that osteoblasts not only secrete VEGF but also serve as important target cells for VEGF in the bone marrow (Helmrich et al., 2013). Correspondingly, overexpression of VEGF led to pre-osteoblast proliferation and increased bone mass, showing again that VEGF could directly act on osteoprogenitors without its angiogenic functions (Maes et al., 2010). Intracellular VEGF signaling, but not exogenous VEGF, was proven to regulate the differentiation of mesenchymal stem cells by inhibiting adipogenesis (Liu et al., 2012; Berendsen and Olsen, 2014). In addition, loss of intracellular VEGF caused increased bone marrow adiposity and bone loss (Berendsen and Olsen, 2014). VEGF also regulated extracellular matrix (ECM) composition through its angiocrine function and thus had significant impact on the distribution of the bone marrow cells (Di Maggio and Banfi, 2022). It is noteworthy that VEGF does not always facilitate osteogenesis. At high concentrations, VEGF can hinder osteogenesis, stimulate the formation of osteoclasts, and enhance their osteolytic activity, ultimately leading to the development of osteoporosis (Nakagawa et al., 2000; Yang et al., 2008; Helmrich et al., 2013). This complex phenomenon observed with VEGF may be attributed to the temporal–spatial effect of its normal distribution and also the highly varied origin and target cells of VEGF. Under physiological conditions, the spatial distribution of VEGF in the bone marrow is determined by a concentration gradient. Therefore, the formation of blood vessels, osteogenesis, and osteoclastic activities are directed toward specific areas of the bone, allowing for bone metabolism to occur in a localized manner. However, too high or too low VEGF will destroy the concentration gradient under the physiological condition, resulting in an imbalance between osteogenesis and osteoclast activity, finally causing bone destruction (Ruhrberg et al., 2002; Gianni-Barrera et al., 2020).

#### PDGF-BB

PDGF-BB is a chemotactic cytokine that promotes the proliferation and differentiation of endothelial progenitor cells and mesenchymal stem cells in the bone marrow (Fiedler et al., 2004; Wang et al., 2012). Notably, Xie et al. discovered that pre-osteoclasts are the primary source of PDGF-BB secretion in the bone marrow (Xie et al., 2014). PDGF-BB facilitates the proliferation and migration of endothelial cells and mesenchymal stem cells through its interaction with the β-type PDGF receptor (PDGF receptor-β), leading to the activation of mitogen-activated protein kinases (MAPKs) and protein kinase B (PKB and Akt) (Xie et al., 2014). Finally, these factors lead to an increase in the number of type H vessels and osteoblasts in the region responsible for bone formation (Xie et al., 2014). Gao et al. discovered that tartrate-resistant acid phosphatase-positive (TRAP+) cells located on the cortical bone have the ability to stimulate the migration of bone marrow mesenchymal stem cells toward the periosteum (Gao et al., 2019). This migration is accompanied by the expression of periostin, which is facilitated by the secretion of PDGF-BB (Gao et al., 2019). PDGF-BB released by TRAP + cells has the potential to stimulate the periosteum of type H vessels and enhance osteogenic activity (Gao et al., 2019). Importantly, bone-derived PDGF-BB was recently found to mediate arterial stiffening and calcification in the brain, suggesting a central role of skeleton-derived PDGF-BB in vascular diseases (Santhanam et al., 2021; Wang et al., 2023).

#### SLIT-3

The SLIT family is a molecule with the neuron guidance function found in the central nervous system and includes three homologous subtypes: SLIT-1, SLIT-2, and SLIT-3 (Long et al., 2004). SLIT-3 is expressed in a variety of tissues and has been linked to angiogenesis and stem cell function (Paul et al., 2013; Qiu et al., 2015). Several studies have explored the role of SLIT-3 in bone tissue, demonstrating its ability to enhance the formation of type H vessels and increase bone mass (Kim et al., 2018; Xu et al., 2018). Additionally, Xu et al. discovered that osteoblasts are the primary source of SLIT-3 secretion. The knockout of SLIT-3 in mouse osteoblasts resulted in a reduction of type H vessels, impaired osteoblast function, and decreased osteogenic activity. Exogenous injection of SLIT-3 can increase the number of type H vessels, accelerate fracture healing, and alleviate osteoporosis (Xu et al., 2018). However, Kim et al. found that SLIT-3 can be secreted by osteoclasts and that SLIT-3 could promote the proliferation and migration of osteoblasts. The knockout of SLIT-3 in mouse osteoclasts resulted in an observed increase in osteoclasts, as well as a decrease in type H vessels and bone mass (Kim et al., 2018). The aforementioned studies have shown that the source of SLIT-3 in the bone is controversial, but targeting SLIT-3 can increase the number of type H vessels and promote osteogenic activity.

#### Notch

The Notch signaling pathway in vascular endothelial cells has angiogenic and osteogenic functions. During angiogenesis, a small number of endothelial cells activated by VEGF will become the tip cells that lead to the growth of blood vessels. Meanwhile, VEGF activates the Notch pathway of the tip cells adjacent to the endothelial cells by upregulating the Notch ligand Dll4, producing stalk cells and completing the process of angiogenesis (Ramasamy et al., 2014; Wang et al., 2017). Studies have shown that the Notch signaling pathway in bone marrow vascular endothelial cells promotes the production of type H vessels, while inhibition of the Notch signaling pathway impairs angiogenesis and osteogenesis (Ramasamy et al., 2014). The effect of the Notch signaling pathway on osteogenesis mainly stems from its promotion of the expression of Noggin in endothelial cells. Noggin inhibits the BMP signaling pathway in pre-osteoblasts expressing osterix, thereby promoting the proliferation and differentiation of osteoblasts (Ramasamy et al., 2014). Additionally, mechanical stimulation influences the activation of the Notch pathway. High blood flow can stimulate Notch signaling, whereas a low blood flow reduces the Notch signaling pathway. Reduced blood flow in aged mice has been demonstrated to result in a decrease in type H vessels and in the development of osteoporosis. Surprisingly, it has been found that activation of the Notch pathway or increasing the skeletal blood flow using the bisphosphonate drug alendronate can reverse the vascular and bone loss phenotype (Ramasamy et al., 2016). Zinc-finger E-box-binding homeobox 1 (ZEB1) is a significant zinc finger transcription factor *in vivo*. Researchers have conducted an investigation into the expression of ZEB1 in type H vessels within the bone. It has been found that ZEB1 plays a crucial role in regulating the expression of Dll4 and Notch signaling through the regulation of H3K4Ac, H3K14Ac, and H3K18Ac. Exogenous ZEB1 can increase type H vessels and treat osteoporosis (Fu et al., 2020).

#### Other contributing factors

Angiogenesis is closely related to osteogenic activity. Therefore, numerous cytokines exhibit the capacity for bidirectional secretion and regulation. Type H vessels are known to express various growth factors, including transforming growth factor β (TGFβ)1, TGFβ3, PDGF-A, and fibroblast growth factor (FGF). These growth factors play crucial roles in regulating osteoblast-related cells (Wan et al., 2010). Meanwhile, established vital pathways during embryogenesis and bone modeling could have important impact on the formation of type H vessels, including Hedgehog, WNT, BMP, FGF, EGF, IGF, and mTORC pathways, via a local or systematic effect (Huang et al., 2016; Zhu et al., 2020). For example, epidermal growth factor-like protein 6 (EGFL6) is an angiogenesis factor that promotes endothelial cell proliferation and migration. Notably, EGFL6 was among the most dominantly expressed proteins during late-stage osteoblast differentiation, and EGFL6 deficiency decreases type H vessel and bone formation during bone repair (Chen et al., 2021). Nephronectin (NPNT), a homolog of EGFL6, was also proven to regulate angiogenesis and osteogenesis *in vitro*, highlighting that osteoblast-originated epidermal growth factors may function as significant regulators of bone marrow angiogenesis (Kuek et al., 2016). The classic osteoclast induction pathway RANK/RANKL was also found to regulate vascular cell survival and proliferation through PI3K and Akt pathways (Kim et al., 2003). RANK/RANKL secreted by type H vessels also supports the aforementioned VAOs (Romeo et al., 2019). Liu et al. found that osteoclasts maintain type H vessels by secreting angiogenin, and decreased angiogenin led to the loss of type H vessels and bone mass (Liu et al., 2021). Nerves usually accompany blood vessels, and nerve growth factors can promote angiogenesis. Tropomyosin-receptor kinase A (TrkA) is a nerve growth factor receptor that activates TrkA on the surface of endothelial cells to directly promote angiogenesis (Lu et al., 2018). The interaction between the nerve growth factor (NGF) and TrkA in sensory terminals has been found to facilitate the release of angiogenic neuropeptides. These neuropeptides bind to NK1 and CGRPR receptors located on the surface of endothelial cells, thereby inducing angiogenesis (Lu et al., 2018). Sema3A, another nerve growth factor, has been demonstrated to inhibit osteoclasts and enhance osteoblast proliferation through the activation of the neuropilin-1 receptor. Thus, it has been observed that Sema3A has the potential to enhance the process of osteogenesis (Hayashi et al., 2012; Fukuda et al., 2013). Addition of silicon to engineered materials could promote bone formation though Sema3A secreted by nerve endings (Ma et al., 2022). Vascular endothelial cells have the ability to secrete significant quantities of Sema3A, which can enhance their own functionality. However, the specific role of Sema3A secreted by endothelial cells in bone remains unclear. Exosomes are a type of extracellular vesicles that are generated by cells. As a crucial component of intercellular communication, extracellular vesicles play a significant role in the transport of nucleic acids, proteins, lipids, and metabolites. They facilitate intercellular information transmission through various mechanisms, including the ligand–receptor interaction, membrane fusion, and endocytosis. Recent studies have demonstrated that non-coding RNA molecules present in exosomes, including miR-210, lncRNA MEG3, and lncRNA H19, play a significant role in the regulation of angiogenesis (Su et al., 2015; Xie et al., 2019; Behera et al., 2021). Sex hormones were also found to be vital regulators of bone blood vessels and might explain sex specificity in bone metabolism. For instance, estrogen was found to regulate lipid use and FA uptake of the bone blood endothelium. Low estrogen levels were associated with accumulated lipid peroxides (LPOs) and accelerated vascular aging, while inhibition of LPO generation improved bone health in aged mice (Rodrigues et al., 2022).

### The role of type H vessels in bone diseases

**FIGURE 2:**
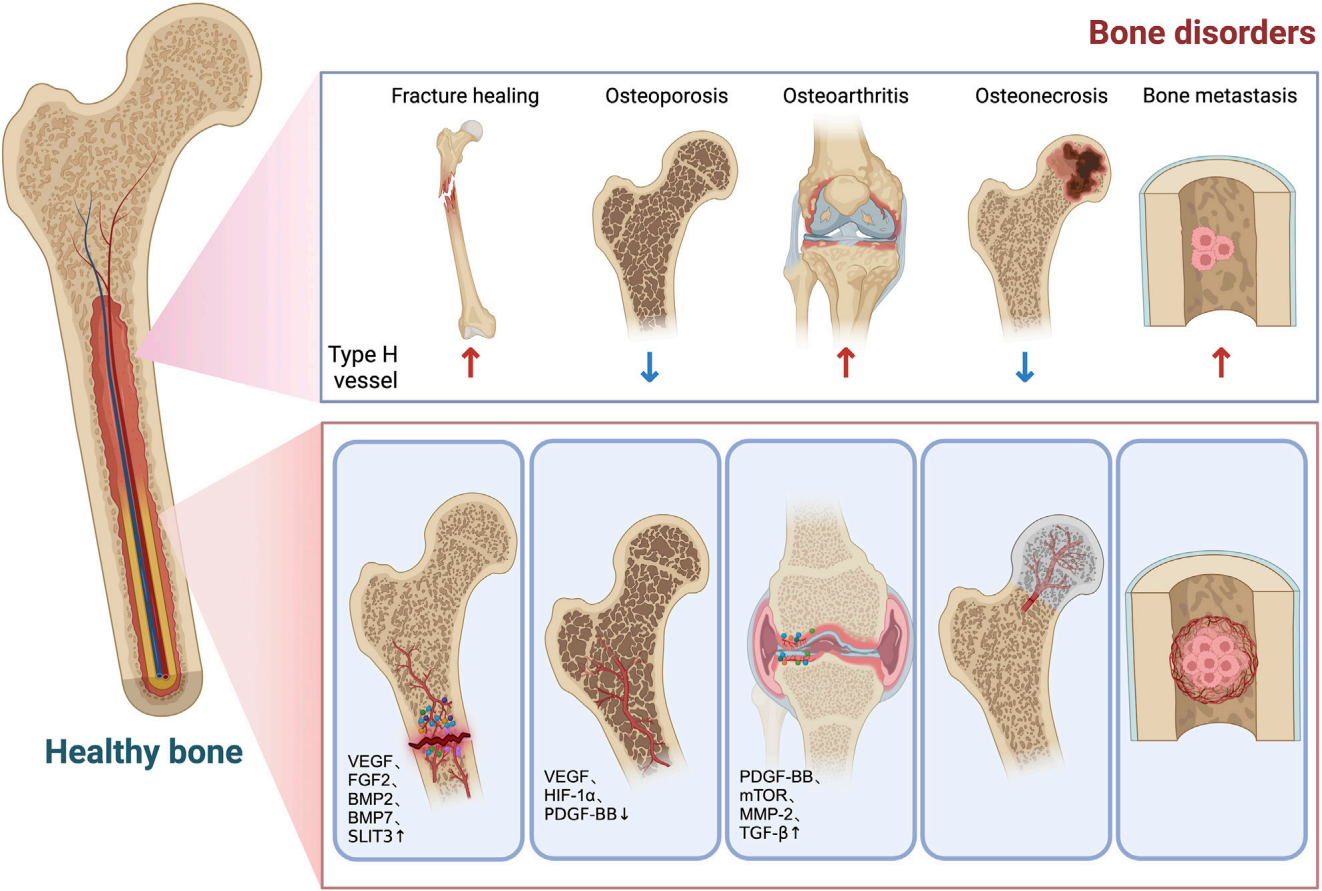
Role of type H vessels in bone disorders. Decreased type H vessel is associated with bone loss and increased fracture risk. After fracture, type H vessels are formed in the callus in response to increased factors (VEGF, FGF2, BMP2, BMP7, SLIT3, etc.) to facilitate bone formation. Type H vessels are decreased in osteoporosis conditions due to decreased HIF-1α/VEGF and PDGF-BB. In osteoarthritis, type H vessel formation in the subchondral bone causes aberrant bone formation and bone marrow sclerosis, which leads to cartilage degeneration. PDGF-BB, mTOR, MMP-2, and TGF-β are involved in the infiltration and overgrowth of type H vessels in osteoarthritis. Decreased blood supply to the femoral head caused partly by diminished type H vessels contributes to development of osteonecrosis. Metastasis tumor cells in bone type L vessels tend to seed in the bone marrow microenvironment. Type H vessels support tumor cell proliferation by providing oxygen, nutrients, and growth factors. During radiation and chemotherapy, increased type H vessels and surrounding pericytes generate a quiescent microenvironment, which impairs treatment outcomes.

The dysregulation of osteoblast–osteoclast activity can give rise to a range of bone disorders. For instance, an imbalance between osteoblasts and osteoclasts can result in the development of osteoporosis, a deficiency in osteogenesis can impede proper fracture healing, and an excessive amount of osteogenesis can contribute to the onset of osteoarthritis. Type H blood vessels have been demonstrated to exhibit associations with a diverse range of bone diseases, which will be discussed in subsequent sections (Figure 2).

#### Fracture healing

After a fracture, the bone tissue sustains damage and the local blood flow is disrupted, resulting in the exudation of inflammatory cells and the formation of hematoma. Subsequently, the blood vessels originating from the bone marrow, cortical bone, and periosteum will gradually extend toward the site of fracture. Following hematoma formation, fibroblasts and chondrocytes will undergo a process of cartilage callus formation, leading to the eventual development of a new bone (Bolander, 1992). Increased neovascularization is essential for fracture healing. Research reported that the use of VEGF could accelerate vascularization and fracture healing. Antagonizing VEGF or its receptor VEGFR1 reduced the number of new blood vessels and the mineralization of fracture callus, ultimately slowing down fracture healing (Street et al., 2002; Kleinheinz et al., 2005). FGF2, BMP2, and BMP7 have also been shown to promote angiogenesis and bone repair (Sivaraj and Adams, 2016). Xu et al. found that injection of SLIT3 could facilitate fracture healing in mice by increasing type H vessels (Xu et al., 2018). Low-frequency pulsed ultrasound has been shown to promote the increase in blood vessels after spinal fusion and improve prognosis (Xu et al., 2016).

#### Osteoporosis

Osteoporosis is characterized by the deterioration of bone tissue resulting from an imbalance between the formation of a new bone (osteogenesis) and the breakdown of an existing bone (osteoclastic activity). Wang et al. found that type H vessels can serve as a reliable indicator for assessing bone mass in the elderly population (Wan et al., 2010). Previous research has consistently shown that the presence of type H vessels decreases to varying extents in individuals with osteoporosis resulting from menopause, glucocorticoid use, and advanced age (Kusumbe et al., 2014; Yang et al., 2018; Li et al., 2020). The decline in type H vessels in individuals with osteoporosis is directly correlated with the reduction in angiogenic factors. Research studies have demonstrated that both menopausal factors and glucocorticoids could decrease the production of PDGF-BB by downregulating the population of pre-osteoclasts, leading to a decline in type H vessels. The augmentation of pre-osteoclasts led to an increase in the production of PDGF-BB, subsequently resulting in the enhancement of type H vessels and bone mass (Yang et al., 2018). Angiogenin, which was derived from osteoclasts, played a crucial role as an angiogenic factor in the maintenance of blood vessels. The reduction in osteoclasts induced by glucocorticoids resulted in a decrease in angiogenin levels and subsequently led to the senescence of blood vessels (Liu et al., 2021). Of note, in grown mice, glucocorticoids caused bone marrow adipocyte senescence, which could cause secondary senescence of bone blood vessels and subsequent bone loss (Liu et al., 2023a). VHL (von Hippel–Lindau)-E3 ubiquitin protein ligase is a crucial protein involved in the regulation of HIF-1α stability. Knocking out the von Hippel–Lindau (VHL) gene in osteoblasts has been shown to upregulate the expression of VEGF and increase type H vessels and bone mass in mice subjected to ovariectomy. In aging mice, the targeted deletion of VHL in vascular cells has been shown to enhance the preservation of type H vessels and increase bone mass. These results suggested that augmenting angiogenic factors could be a potential approach for treating osteoporosis (Hu and Olsen, 2016). Gao et al. found that the local administration of tetramethylpyrazine (TMP) rescued vascular proliferation and bone volume through the AMPK-mTORC-HIF1-α signaling pathway in aging mice (Gao et al., 2018). It is worth noting that certain rehabilitation therapies, such as electromagnetic pulse, as well as traditional Chinese medicine preparations like Gushukang and harmine, have also demonstrated the ability to alleviate osteoporosis by promoting blood vessel formation. The findings suggested that the upregulation of type H vessels could potentially serve as a mechanism in the treatment of osteoporosis through rehabilitation or traditional Chinese medicine (Huang et al., 2018; Li et al., 2020; Wang et al., 2022).

#### Osteoarthritis

Osteoarthritis is a prevalent joint disease among individuals in middle-aged and elderly populations. It is primarily characterized by the degeneration and deterioration of cartilage, as well as the abnormal vascularization and osteogenesis of the subchondral bone (Hunter and Bierma-Zeinstra, 2019). Among the various pathological changes observed in osteoarthritis, angiogenesis, abnormal hyperemia, and bone marrow edema of the subchondral bone play significant roles in disease development (Hu et al., 2021). Mapp et al. conducted a study that demonstrated the occurrence of subchondral bone angiogenesis in the early stages of osteoarthritis, leading to the development of subchondral bone sclerosis and osteophyte formation (Mapp and Walsh, 2012). Additionally, Cui et al. observed an increase in type H vessels in mouse models of osteoarthritis. The excessive proliferation of type H vessels in the subchondral bone and subchondral bone sclerosis can be mitigated by the traditional Chinese medicine halofuginone through the inhibition of MMP-2 and TGF-β (Kleinheinz et al., 2005). Lu et al. conducted a study to investigate the relationship between the activation of mammalian targets of the rapamycin complex (mTOR) in chondrocytes and the secretion of VEGF, as well as the formation of type H vessels in the subchondral bone. Inhibition of the mTOR pathway has been shown to have a potential therapeutic effect in reducing the formation of type H vessels and slowing down the progression of osteoarthritis (Lu et al., 2018). Cartilage tissue is usually avascular. In osteoarthritis, certain blood vessels infiltrate the interface between the cartilage and the subchondral bone, thereby invading the cartilage tissue (Mapp et al., 2008). Recent studies have indicated that in the initial stages of mouse arthritis models, there is a notable increase in the secretion of PDGF-BB and the development of type H vessels in the subchondral bone, which is accompanied by nerve growth. Overexpression of PDGF-BB in osteoclasts has been shown to result in subchondral bone vascularization and spontaneous cartilage destruction. Conversely, inhibiting PDGF-BB expression in osteoclasts has been found to improve the arthritis phenotype (Su et al., 2020). Metformin was found to inhibit metabolic-associated osteoarthritis partly though inhibition of type H vessels (Liu et al., 2023b). Type H vessels have the ability to facilitate the resorption of cartilage tissue in juvenile mice through the secretion of MMP-9. This finding suggests a potential novel mechanism for cartilage tissue degradation in osteoarthritis. However, further investigation is required to provide *in vivo* evidence for this hypothesis (Romeo et al., 2019).

#### Femoral head necrosis

Femoral head necrosis refers to the death of bone tissue in the femoral head due to a compromised or disrupted blood supply, resulting in avascular necrosis (Weinstein, 2012). Weinstein et al. discovered that the rate of bone metabolism in the femoral head is higher than that in the distal femur (Weinstein et al., 2017). Due to the significant association between type H vessels and bone metabolism processes, it is plausible to hypothesize that the abundance of type H vessels in the femoral head region is greater than that in the distal femur. However, this hypothesis requires additional verification (Weinstein et al., 2017). Weinstein et al. also demonstrated that glucocorticoids have the potential to induce reduced blood vessel density and fragmentation in the femoral head, thereby exacerbating femoral head edema (Weinstein et al., 2017). However, the administration of parathyroid hormone therapy in the model of steroid-induced femoral head necrosis has been found to increase the blood vessel density in the femoral head, but it does not alleviate femoral head necrosis. It could be posited that the etiology of femoral head necrosis is not solely associated with the reduction of type H vessels (Lane et al., 2018).

#### Tumor bone metastasis

Bone tissue is frequently targeted by tumor cells for metastasis, making it one of the most prevalent sites for such spread. The unique anatomical and physiological characteristics of blood vessels in the bone marrow play a crucial role in the process of tumor bone metastasis. Corresponding to the type H vessels located at the metaphysis on both sides of the long bone, the blood vessels present in the diaphysis are referred to as L-type blood vessels (type L vessel). These vessels are characterized by low expression of EMCN and CD31 and are commonly known as sinusoids (Sivaraj and Adams, 2016). Type L vessels lack perivascular cells. The high permeability of the sinusoidal endothelium is attributed to the lack of tight junctions between endothelial cells. In addition, the large diameters and slow blood flow make type L vessels ideal for frequent exchange of substances and cells. Additionally, these vessels play a crucial role in the homing and maintenance of hematopoietic stem cells. CXCL12 that is expressed by the type L endothelium has the ability to bind to the CXCR4 receptor found on circulating tumor cells. This binding facilitates the attachment of circulating tumor cells to the bone marrow microenvironment, resembling the process of stem cell homing (Bussard et al., 2008; Kusumbe, 2016). In conclusion, the aforementioned attributes of type L vessels contribute to the preservation of the microenvironment of bone marrow cells and facilitate bidirectional transportation of hematopoietic cells. In addition to their role in invasion and planation of circulating tumor cells within the bone marrow microenvironment, type L vessels have been found to contribute to the development of bone metastases (Kusumbe, 2016). It has been observed that tumor cells have a higher tendency to localize in areas where type L vessels are present, as opposed to type H vessels. However, it is worth noting that type H vessels exhibit a higher level of metabolic activity and proliferation. The role of type H vessels in tumor cells is significant as they transport oxygen, nutrients, cells, and growth factors (Ghajar et al., 2013; Kusumbe, 2016). Studies have demonstrated that reducing the volume of type H vessels in the bone marrow can enhance the sensitivity of breast cancer bone metastases to chemoradiotherapy. In a more recent work, Singh et al. showed that type H-associated PDGFRβ^+^ pericytes maintained the quiescent microenvironment in the bone marrow, which was compromised in aging conditions (Singh et al., 2019). Radiation or chemotherapy promoted proliferation of type H vessels and pericytes, which further strengthened the quiescence-promoting secretome of these vascular cells, and thereby contributed toward chemoresistance. Interestingly, simply reducing the blood flow of the bone marrow could inhibit pericyte expansion and render cancer cells susceptible to radiation and chemotherapy (Singh et al., 2019).

## Summary

The blood vessels within the bone marrow serve as conduits for the transportation of oxygen, nutrients, and cells. It is the living environment for different bone marrow cell groups. Therefore, blood vessels play a crucial role in bone metabolism. Type H vessels are a subtype of bone marrow vessels discovered in recent years, which play an important role in regulating bone development, osteogenesis, and bone remodeling. Numerous studies have demonstrated that osteoblasts, osteoclasts, and other bone marrow cells establish an intricate regulatory network with blood vessels via various signaling pathways, including HIF-1α, VEGF, PDGF, SLIT-3, and Notch. Therefore, these communications achieve precise regulation of bone metabolism by controlling the formation of new blood vessels. The current treatment methods for bone diseases mainly focus on inhibiting osteoclasts or promoting osteoblast function. The investigation into type H vessels and their interaction with bone marrow cells is anticipated to yield novel insights for bone treatment and establish a stronger theoretical foundation for future therapeutic approaches.
